# Single‐Nucleus RNA Sequencing Reveals that Decorin Expression in the Amygdala Regulates Perineuronal Nets Expression and Fear Conditioning Response after Traumatic Brain Injury

**DOI:** 10.1002/advs.202104112

**Published:** 2022-01-17

**Authors:** Yingwu Shi, Xun Wu, Jinpeng Zhou, Wenxing Cui, Jin Wang, Qing Hu, Shenghao Zhang, Liying Han, Meixuan Zhou, Jianing Luo, Qiang Wang, Haixiao Liu, Dayun Feng, Shunnan Ge, Yan Qu

**Affiliations:** ^1^ Department of Neurosurgery Tangdu Hospital Fourth Military Medical University Xi'an Shaanxi 710038 China; ^2^ School of Biomedical Engineering Shanghai Jiao Tong University Shanghai 200240 China; ^3^ Department of Neurosurgery West Theater General Hospital Chengdu Sichuan 610083 China

**Keywords:** amygdala, decorin, fear conditioning, perineuronal nets, traumatic brain injury

## Abstract

Traumatic brain injury (TBI) is a risk factor for posttraumatic stress disorder (PTSD). Augmented fear is a defining characteristic of PTSD, and the amygdala is considered the main brain region to process fear. The mechanism by which the amygdala is involved in fear conditioning after TBI is still unclear. Using single‐nucleus RNA sequencing (snRNA‐seq), transcriptional changes in cells in the amygdala after TBI are investigated. In total, 72 328 nuclei are obtained from the sham and TBI groups. 7 cell types, and analysis of differentially expressed genes (DEGs) reveals widespread transcriptional changes in each cell type after TBI are identified. In in vivo experiments, it is demonstrated that Decorin (Dcn) expression in the excitatory neurons of the amygdala significantly increased after TBI, and Dcn knockout in the amygdala mitigates TBI‐associated fear conditioning. Of note, this effect is caused by a Dcn‐mediated decrease in the expression of perineuronal nets (PNNs), which affect the glutamate‐*γ*‐aminobutyric acid balance in the amygdala. Finally, the results suggest that Dcn functions by interacting with collagen VI *α*3 (Col6a3). Consequently, the findings reveal transcriptional changes in different cell types of the amygdala after TBI and provide direct evidence that Dcn relieves fear conditioning by regulating PNNs.

## Graphical abstract

Single‐nucleus RNA sequencing is employed to investigate the transcriptional changes of individual cells from the amygdala tissues of sham and traumatic brain injury (TBI) mice. Decorin is significantly increased in excitatory neurons. Decorin knockout in the amygdala could mitigate TBI‐induced fear condition. Of note, high Decorin expression suppresses the expression of perineuronal nets by interacting with collagen VI *α*3.

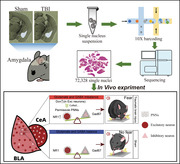

## 1 Introduction

Traumatic brain injury (TBI) is a major cause of long‐term neurological and psychiatric disorders in domestic, sports, and military environments.^[^ ^1^ ^]^ More than 20% of TBI patients suffer from posttraumatic stress disorder (PTSD).^[^ ^2^ ^]^ The amygdala belongs to the limbic system and plays an important role in the formation of fear conditioning and anxiety behavior, which is the neurobiological basis of PTSD.^[^ ^3^ ^]^ Clinical studies have shown that the volume of the amygdala changes significantly after TBI and that this change is related to PTSD.^[^ ^4^, ^5^ ^]^ After TBI, changes in the morphology of neurons in the amygdala and changes in gene expression in these neurons were observed in animal studies.^[^ ^6^, ^7^ ^]^ However, the mechanism by which the amygdala is involved in fear and anxiety after TBI is largely unknown.

Perineuronal nets (PNNs), which surround neurons, both regulate synaptic plasticity and act as a protective barrier.^[^ ^8^ ^]^ Injection of rats with the chondroitin sulfate proteoglycan (CSPG)‐degrading enzyme chondroitinase ABC (chABC) decreased PNNs expression, resulting in increased cortical *γ*‐aminobutyric acid (GABA)ergic neuron plasticity.^[^ ^9^ ^]^ In addition, chABC injection in the basolateral amygdala (BLA) rendered subsequently acquired fear memories susceptible to erasure;^[^ ^10^ ^]^ removal of PNNs disrupts recall of a remote fear memory by affecting synchronized theta oscillations between the secondary visual cortex and BLA.^[^ ^11^ ^]^ PNNs in the ventral hippocampus were found to be closely related to early life adversity‐induced anxiety behavior;^[^ ^12^ ^]^ loss of Ankyrin‐R from forebrain interneurons reduces and disrupts PNNs, and decreases anxiety‐like behaviors.^[^ ^13^ ^]^ Decorin (Dcn) is one of the extracellular matrix (ECM)‐related genes, and previous study has reported that Dcn in hippocampus is related to memory and learning,^[^ ^14^ ^]^ but the role of Dcn in fear and anxiety has not been reported. Furthermore, changes in PNNs expression in the brain after TBI have been widely reported.^[^ ^15^, ^16^, ^17^ ^]^ However, whether fear conditioning and anxiety behavior caused by TBI are related to PNNs remains unclear.

The amygdala, which anatomically consists of the central amygdala (CeA) and BLA, is a highly cellular, heterogeneous brain region that contains excitatory neurons, inhibitory neurons, and various nonneuronal cell types.^[^ ^18^ ^]^ Changes in the structure and function of neurons are closely related to changes in neuronal transcription.^[^ ^19^ ^]^ However, traditional experimental methods such as bulk RNA sequencing (bulk RNA‐seq) cannot be used to investigate cell type‐specific transcription, hindering the discovery of differentially expressed genes (DEGs) in the amygdala after TBI. To overcome this technical barrier, we performed single‐nucleus RNA sequencing (snRNA‐seq) to analyze transcriptional changes in the amygdala after TBI.

Here, we identified different cell types in the amygdala and analyzed DEGs in these cell types between the sham and TBI groups to reveal the mechanism by which PNNs expression changes in TBI and the relationship between fear conditioning and PNNs.

## 2 Results

### 2.1 Identification of the Cell Composition of the Amygdala

**Figure 1:**
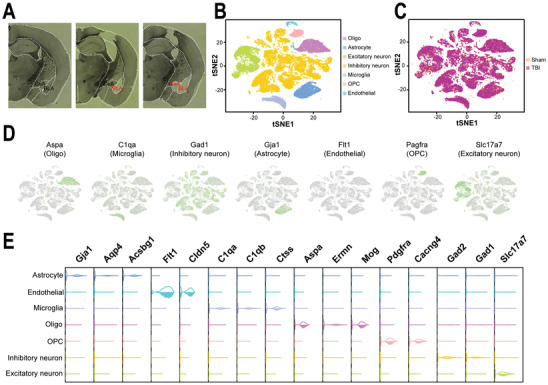
Classification of cell types in the mouse amygdala based on snRNA‐seq data. A) Photomicrographs showing dissection of the BLA and CeA subregions of the amygdala. B) t‐SNE plot showing the different cell types in the amygdala based on transcriptome data. C) t‐SNE plot showing sample types from the sham and TBI groups. D) Expression of marker genes (highlighted with green color) in the cell types on the t‐SNE plot. E) Violin plot showing cell type‐specific gene marker expression in the different cell clusters.

We classified cell types based on their transcriptome features to study the cellular heterogeneity of the amygdala. First, the amygdala was isolated from acute coronal brain sections of TBI and sham mice (**Figure** **1**A). We dissected the CeA and BLA regions, which are known components of the amygdala.^[^ ^18^ ^]^ A total of 79 946 single nuclei from 6 independent biological samples (3 sham samples vs 3 TBI samples) were sequenced in this study. Low‐quality nuclei (please refer to the Experimental Section) were filtered out. Finally, we obtained 72 328 high‐quality nuclei, which were separated into 7 cell types (Figure 1B). Importantly, each of the 7 cell types was detected in each of the sham and TBI samples (Figure 1C). According to the expression of cell type‐specific marker genes, the neurons consisted of inhibitory neurons (Gad1 and Gad2) and excitatory neurons (Slc17a7), and the nonneuronal cells were clustered as astrocytes (Gja1, Aqp4 and Acsbg1), microglia (C1qa, C1qb and Ctss), endothelial cells (Flt1 and Cldn5), oligodendrocytes (Oligos) (Aspa, Ermn and Mog), and oligodendrocyte precursor cells (OPCs) (Pdgfra and Cacng4) (Figure 1D,E).

### 2.2 Neuronal Subtypes in the Amygdala with Distinct Gene Expression

**Figure 2:**
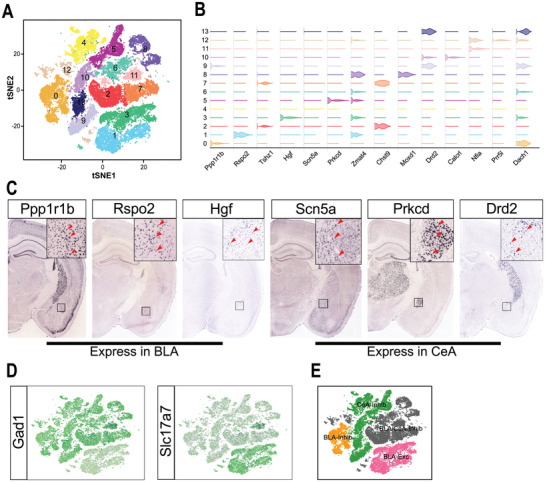
The amygdala contains distinct neuron subtypes. A) t‐SNE plot showing that neurons in the amygdala can be further divided into 14 clusters. B) Violin plot showing the expression of marker genes in the 14 clusters. C) Results of RNA ISH analysis of marker genes in the amygdala. D) Expression of Gad1 and Slc17a7 (highlighted by green color) on the t‐SNE plots. E) The neurons are divided into 4 subtypes on the t‐SNE plots according to the location of marker gene expression (the BLA, CeA, or total) and neuronal properties (inhibitory or excitatory).

Neurons, the largest and most diverse cell population in the amygdala, were classified by morphology, anatomical location, and histological features. Thus, we further clustered the neurons of the amygdala and identified 14 transcriptionally distinct subtypes (**Figure** **2**A). Each neuron subtype was identified by the unique expression of one or a combination of 3–4 marker genes (Figure 2B). First, we observed the results of RNA in situ hybridization (ISH) for these marker genes in the Allen Brain Atlas,^[^ ^20^ ^]^ after which we determined the anatomical locations where these genes were expressed (the BLA, CeA, or total) (Figure 2C; and Figure S1, Supporting Information). The Ppp1r1b, Rspo2, and Hgf, marker genes of clusters 0, 1, and 3, were expressed in the BLA. The Scn5a, Prkcd, and Drd2, marker genes of clusters 4, 5, 9, 10, and 13, were mainly expressed in the CeA. The rest of the marker genes of clusters 2, 6, 7, 8, 11, and 12, such as Tshz1, Moxd1, and Zmat4, were expressed in both the BLA and CeA. In addition, we identified the excitatory and inhibitory properties of these neurons based on Slc17a7 and Gad1 expression (Figure 2D). According to the location of marker gene expression (the BLA, CeA, or total) and their neuronal properties (inhibitory or excitatory), the neurons were divided into 4 subtypes: BLA‐Exc neurons (11 630 neurons), BLA‐Inhib neurons (7391 neurons), CeA‐Inhib neurons (13 264 neurons), and CeA/BLA‐Inhib neurons (10 980 neurons) (Figure 2E).

### 2.3 TBI Alters Gene Expression in Different Cell Types

**Figure 3:**
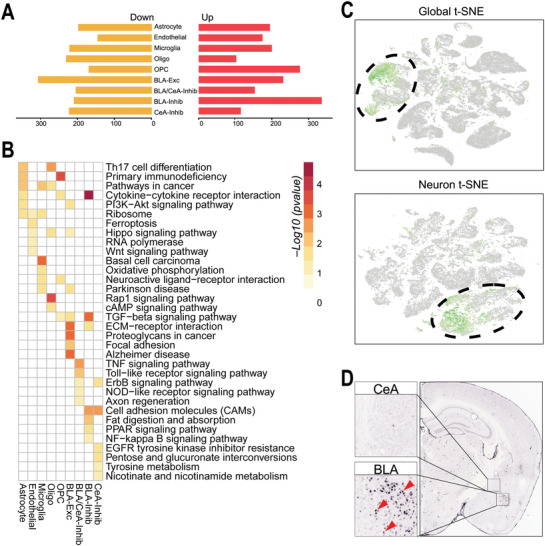
Broad transcriptional differences in each amygdala cell type between the sham and TBI groups. A) Numbers of up‐ and downregulated genes in the different cell types. B) Heatmap showing representative enriched functional pathways based on the DEGs of the different cell types. C) Expression of Dcn (highlighted by green color) in all cells and the neuron subtypes on the t‐SNE plots. D) The results of RNA ISH analysis showing the expression of Dcn mainly in the BLA.

To investigate the specific genes and pathways that cause TBI pathogenesis in different cell types of the amygdala, we identified DEGs between the two groups. With a cutoff of 0.05 FDR and |log2FC|>1 (the “MAST” statistical framework,^[^ ^21^ ^]^ Benjamini & Hochberg‐corrected) were used as criteria, and the expression of a maximum of 333 genes and a minimum of 102 genes was altered across the different cell types (**Figure** **3**A). In addition, Kyoto Encyclopedia of Genes and Genomes (KEGG) analysis of the DEGs was performed to investigate how these transcriptional changes affect the functions of the various cell types, and the top‐ranked pathways are listed in Figure 3B. Our results showed that in cell types of the amygdala, diverse pathways are involved in TBI: Th17 cell differentiation, primary immunodeficiency, and pathways in cancer were related to astrocytes; the ribosome, ferroptosis and Hippo signaling pathways were altered in endothelial cells; and basal cell carcinoma, oxidative phosphorylation, and neuroactive ligand receptor interactions were related to oligos. Furthermore, primary immunodeficiency, cytokine‐cytokine receptor interactions and the TGF‐beta signaling pathway were altered in OPCs; ECM receptor interactions, proteoglycans in cancer, and focal adhesion were altered in BLA‐Exc neurons; and the TNF signaling pathway, Toll‐like receptor signaling pathway and ErbB signaling pathway were altered in BLA/CeA‐Inhib neurons. Additionally, cell adhesion molecules, fat digestion and absorption, and the PPAR signaling pathway were altered in BLA‐Inhib neurons, and EGFR tyrosine kinase inhibitor resistance, pentose, and glucuronate interconversion and tyrosine metabolism were altered in CeA‐Inhib neurons. Decorin (Dcn), the top DEG in excitatory neurons, was found to be specifically expressed in BLA‐Exc neurons (Figure 3C). In addition, ISH data showed that Dcn was expressed in the BLA but not in the CeA (Figure 3D), which is consistent with snRNA‐Seq data. The violin plot showed that Dcn was mainly express in excitatory neurons, but not expressed or little expressed in other cell types (Figure S2A, Supporting Information). Furthermore, double‐labeling IF of Dcn and cell markers were performed, and the results showed that Dcn mainly colocalized with CaMKII (an excitatory neurons marker) (Figure S2B, Supporting Information). The top DEGs in other cell types (see in Table S1, Supporting Information), such as Tac1 and Ttr, were expressed in more than one cell type (Figure S3, Supporting Information). In addition, we performed RT‐qPCR, which confirmed changes in the expression of the DEGs in the different cell types (Figure S4, Supporting Information). The expression of most of these genes was upregulated in the TBI group, but there were some genes whose expression did not change; these results may be explained by the fact that these cell types account for too small a proportion of the total, and further fluorescence in situ hybridization (FISH) is needed to analyze this difference. Dcn is a prototype small leucine‐rich proteoglycan that consists of a glycosaminoglycan (GAG) chain and core protein. Previous studies reported that Dcn is an ECM‐related gene that can suppress the expression of CSPGs and delay fiber formation.^[^ ^22^, ^23^ ^]^ Both CSPGs and fiber are main components of PNNs. We speculate that Dcn may regulate synaptic plasticity by decreasing the expression of PNNs in the amygdala and affect TBI‐induced anxiety behavior or fear conditioning. For these reasons, we focused on Dcn in the following experiments.

### 2.4 Verification that Dcn Expression is Upregulated in the Amygdala after TBI

**Figure 4:**
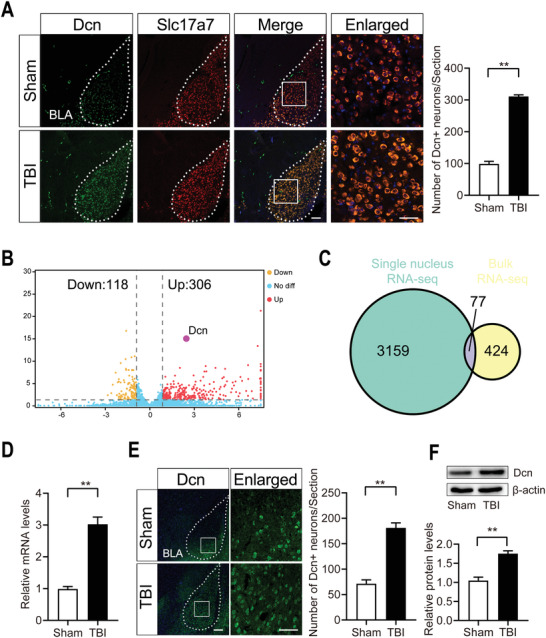
Validation that Dcn expression is upregulated in the BLA after TBI. A) Representative FISH micrographs showing that Dcn expression in the BLA was upregulated in the TBI group compared to the sham group. Scale bar, 100 µm; enlarged: scale bar 100 µm. Data are presented as the mean ± SEM, *n* = 3, **p < 0.01 (*t*‐test). B) Volcano plot showing that Dcn is one of the upregulated DEGs in the TBI group compared to the sham group identified by bulk RNA‐seq. C) Venn diagram showing that more DEGs were captured by single nucleus RNA‐seq than by bulk RNA‐seq. D) RT‐qPCR results showing that Dcn expression was upregulated after TBI. Data are presented as the mean ± SEM, *n* = 3, **p<0.01 (*t*‐test). E) Immunofluorescence analysis showing the number of Dcn+ neurons in the BLA in sham and TBI group. Scale bar, 100 µm. Data are presented as the mean ± SEM, *n* = 3, **p<0.01 (*t*‐test). F) Western blotting results showing the increase in Dcn at the protein level after TBI in the BLA. Data are presented as the mean ± SEM, *n* = 3, **p<0.01 (*t*‐test).

We next aimed to validate the snRNA‐seq results, which showed a significantly increase in Dcn expression in the TBI group compared to the sham group. First, we used FISH to assess the colocalization of Dcn with the corresponding excitatory neuron marker gene slc17a7 in the BLA. The results showed that in the BLA, Dcn was synthesized by excitatory neurons, and its expression was significantly upregulated after TBI (**Figure** **4**A). In addition, we performed bulk RNA‐seq analysis of the amygdala of sham and TBI mice to independently verify the snRNA‐seq results. The results showed that Dcn was one of the DEGs identified by bulk RNA‐seq (Figure 4B), and Venn diagram analysis showed that the expression of 77 genes was changed according to both the bulk RNA‐seq and snRNA‐seq results (Figure 4C). The RT‐qPCR results showed that Dcn mRNA levels were significantly increased after TBI in the amygdala (Figure 4D). Finally, we observed through immunofluorescence (IF) analysis and Western blotting that the protein expression level of Dcn substantially increased after TBI in the BLA (Figure 4E,F).

### 2.5 Dcn Knockout Alleviates the TBI‐Associated Fear Conditioning Response by Promoting PNNs Expression

**Figure 5:**
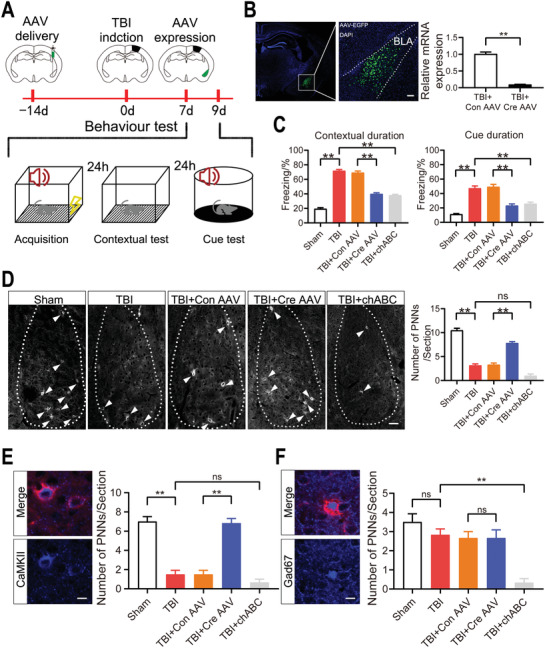
Dcn knockout increased the expression of PNNs and attenuated TBI‐induced fear conditioning. A) Timeline of the experimental design. Before CCI, AAV was injected into the BLA of Dcn^flox/flox^ mice to knock out Dcn, and the mice were then used for subsequent experiments. B) Quantification of Dcn mRNA levels in animals injected with cre AAV or control AAV. Data are presented as the mean ± SEM, *n* = 3, **p<0.01 (*t*‐test). C) Contextual and cued fear conditioning analyses of the 5 groups. Data are presented as the mean ± SEM, *n* = 6, **p<0.01 (one‐way ANOVA with Bonferroni's post hoc test). D) Representative stained images showing PNNs expression in the BLA of the 5 groups. Scale bar, 100 µm. Data are presented as the mean ± SEM, *n* = 6, **p<0.01 (one‐way ANOVA with Bonferroni's post hoc test). E) The numbers of PNNs+CaMK II+ neurons in the BLA in the different groups. Scale bar, 10 µm. Data are presented as the mean ± SEM, *n* = 6, **p<0.01 (one‐way ANOVA with Bonferroni's post hoc test). F) The numbers of PNNs+Gad67+ neurons in the BLA in the different groups. Scale bar, 10 µm. Data are presented as the mean ± SEM, *n* = 6, **p<0.01 (one‐way ANOVA with Bonferroni's post hoc test).

The amygdala is a core brain region that processes fear and anxiety; thus, we identified the function of Dcn in fear conditioning and anxiety behavior by knocking out Dcn in vivo in AAV‐hSyn‐cre‐EGFP and Dcn^flox/flox^ mice (**Figure** **5**A). 3 weeks after AAV injection, RT‐qPCR result showed that the expression of Dcn in BLA significant decreased in Cre AAV injection group (Figure 5B). First, the results of a fear conditioning response (FCR) showed that Dcn knockout attenuated TBI‐induced contextual and cued fear responses, and the changes were the same as those observed upon chABC injection (Figure 5C). However, there was no difference in the elevated plus maze (EPM) test and open field (OF) test results between the five groups (Figure S5, Supporting Information). To investigate the molecular mechanism of Dcn, we assessed the levels of PNNs in the BLA. The IF results showed that TBI decreased the expression of PNNs compared to that in the sham group. In TBI mice, chABC injection would not affect the PNNs expression in BLA, while Dcn knockout increased the expression of PNNs compare to the control AAV injection group (Figure 5D). Interestingly, in the BLA of TBI mice, there was no difference in the expression of PNNs after chABC injection, but the FCR test results showed completely opposite findings between the two groups. Besides, Unlike the cortex, in which PNNs surround only inhibitory neurons, in the BLA, PNNs surround inhibitory and excitatory neurons.^[^ ^24^ ^]^ Whether Dcn only increased PNNs around excitatory neurons or all PNNs is unclear. Thus, we assessed whether the numbers of excitatory and inhibitory neurons surrounded by PNNs changed. Compare to the sham group, TBI only decreased the number of PNNs surrounding excitatory neurons (PNNs+CaMKII+) while did not change the number of PNNs surrounding inhibitory neurons (PNNs+Gad67+) in the BLA. The results showed that in the BLA of TBI mice, after chABC injection the number of PNNs+Gad67+ neurons was decreased, while PNNs+CaMKII+ neurons was unchanged compare to the TBI group; after Dcn knockout the number of PNNs+CaMKII+ was decreased compare to the control AAV injection group, while the number of PNNs+Gad67+ was unchanged between the two groups (Figure 5E,F).

### 2.6 Dcn Knockout Could Restore the TBI‐Associated Glutamate‐GABA Imbalance

**Figure 6:**
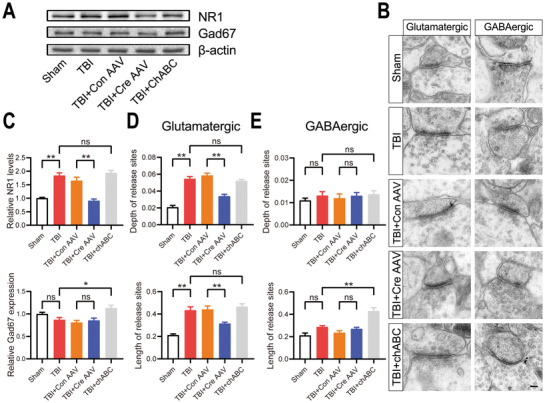
Dcn knockout maintained the glutamate‐GABA balance in the BLA after TBI. A,C) Western blotting results showing the expression levels of NR1 and Gad67 in BLA tissue. Data are presented as the mean ± SEM, *n* = 6, **p<0.01, *p<0.05 (one‐way ANOVA with Bonferroni's post hoc test). B) TEM image of excitatory neuron and inhibitory neuron synapses in the BLA in the different groups. Scale bar, 100 nm. D) The depths and lengths of postsynaptic densities in the glutamatergic neurons. Data are presented as the mean ± SEM, *n* = 50 synapses from 10 mice, **p<0.01 (one‐way ANOVA with Bonferroni's post hoc test). E) The depths and lengths of postsynaptic densities in the GABA neurons. Data are presented as the mean ± SEM, *n* = 50 synapses from 10 mice, **p<0.01 (one‐way ANOVA with Bonferroni's post hoc test).

We observed the expression levels of Gad67 and NR1, which are indicative of GABA and *N*‐methyl‐*D*‐aspartate (NMDA) receptor levels, respectively, by Western blotting. NR1 levels in the BLA were increased while Gad67 levels were unchanged after TBI compare to the sham group. In the BLA of TBI mice, the results showed that after chABC injection Gad67 levels were increased while NR1 levels were unchanged compare to the TBI group; after Dcn knockout NR1 levels were decreased while Gad67 levels were unchanged compare to the control AAV injection group (**Figure** **6**A,C). The expression level of NR2(NR2A, NR2B) and NR3(NR3A, NR3B), which are also subunits of NMDA receptors, were observed in the different groups. The Western blotting analysis revealed that after TBI the expression of NR2A and NR2B were significantly increased compared to sham group; in the BLA of TBI group mice, after Dcn knockout NR2A and NR2B levels were significant decreased but no changes were observed after chABC injection. While after TBI the expression of NR3A and NR3B were unchanged compared to sham group; in the BLA of TBI group mice, NR3A and NR3B were unchanged after Dcn knockout or chABC injection (Figure S6, Supporting Information). Besides, transmission electron microscopy (TEM) was performed to observe glutamatergic and GABAergic synapses in the BLA. Glutamatergic terminals were identified by the presence of thick postsynaptic densities and round synaptic vesicles, while GABAergic synapses were identified by the presence of symmetric release sites and oval synaptic vesicles.^[^ ^25^, ^26^ ^]^ Compared to the sham group, TBI induced substantial increases in the mean length and depth of excitatory synapses in the BLA, while no difference was observed of inhibitory synapses between the two groups (Figure 6B,D,E). In the BLA of TBI mice, we observed a significant increase in the mean length of inhibitory synapses after the chABC injection compare to TBI group, while no difference was observed of excitatory synapses between the two groups; after Dcn knockout, we observed a substantial decrease in the mean length and depth of excitatory synapses compare to the control AAV injection group, while no difference was observed of inhibitory synapses between the two groups (Figure 6B,D,E). These results suggested that Dcn is synthesized by excitatory neurons and that the expression level increases after TBI. High expression of Dcn could specifically decrease the number of PNNs surrounding excitatory neurons and result in hyperexcitability in the BLA, ultimately leading to increased fear conditioning response.

### 2.7 The Col6a3 Protein Physically Interacts with Dcn

**Figure 7:**
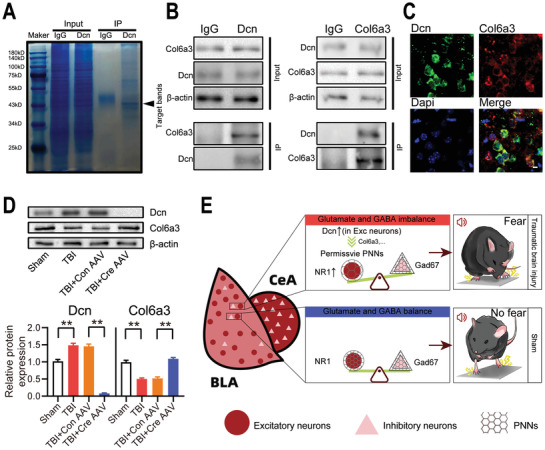
The Col6a3 protein binds Dcn. A) IP samples were run on SDS‐PAGE gels and then stained with Coomassie Brilliant Blue. B) Results of co‐IP analysis confirming the interaction between Dcn and Col6a3. C) IF micrographs showing the colocalization of Dcn and Col6a3 in the mouse BLA. Scale bar, 10 µm. D) Western blotting analysis of the expression of Dcn and Col6a3 in the mouse BLA of the TBI and Dcn‐knockout groups. Data are presented as the mean ± SEM, *n* = 6, **p<0.01 (one‐way ANOVA with Bonferroni's post hoc test). E) Graphical abstract for the study.

Liquid chromatography coupled to tandem mass spectrometry (LC–MS/MS) was used to detect the proteins that physically interact with Dcn and elucidate the possible molecular mechanism by which Dcn suppresses PNNs expression. As shown in **Figure** **7**A, a distinct band corresponding to ≈40 kDa was observed in the product immunoprecipitated by an anti‐Dcn antibody. The immunoprecipitated and lgG bands were then subjected to subsequent LC‐MS/MS analysis; the results showed that collagen VI *α*3 (Col6a3) and collagen XVII *α*1 (Col17a1) physically interact with Dcn and are components of the ECM. Coimmunoprecipitation (co‐IP) in HT22 cells and IF assays in the mouse BLA tissues showed a physical interaction between Dcn and Col6a3 (Figure 7B,C). However, no physical interaction between Dcn and Col17a1 was observed. The Western blotting results showed that the expression of Col6a3 was decreased in the BLA after TBI compare to the sham group; while in the BLA of TBI mice, Dcn knock out could increase the expression of Col6a3 (Figure 7D). In summary, we propose that after TBI, Dcn induces the upregulation of PNNs through Col6a3 and causes glutamate‐GABA imbalance in the BLA, ultimately resulting in augmented fear conditioning response (Figure 7E).

## 3 Discussion

In this study, we first used snRNA‐seq to identify the different cell types and their unique transcriptional features in the mouse amygdala. Analysis of DEGs between the sham and TBI groups showed that gene transcription in each cell type underwent widespread changes after TBI. ECM‐related pathways in excitatory neurons in the BLA underwent a significant change, and among the DEGs, Dcn attracted our attention due to its unique expression pattern and novel clustering. The upregulation of Dcn expression in the BLA after TBI was verified in vivo. Then, we knocked out the expression of Dcn in the mouse BLA; behavioral testing showed that TBI‐induced FCR were reduced, and morphological analysis indicated that in the BLA of TBI mice, the expression of PNNs was increased by Dcn knockout. We inferred that PNNs influence the glutamate‐GABA balance, which is the basis for maintaining the normal function of the BLA. Finally, we investigated proteins that physically interact with Dcn. Our research provides insight into the molecular mechanism of TBI‐induced fear conditioning response.

The amygdala exhibits rich cellular heterogeneity; similar to other snRNA‐seq studies of different brain regions,^[^ ^27^ ^]^ we identified 7 cell types in this study. The CeA and BLA were included in this study because of their important functions in fear and anxiety.^[^ ^28^ ^]^ Neurons were further classified into 14 clusters, which were identified using distinctive markers. Some marker genes have been reported in previous studies; for example, Prkcd and Drd2 have been studied in the CeA,^[^ ^29^, ^30^ ^]^ and Ppp1r1b and Rspo2 in the BLA play an important role in fear memory.^[^ ^31^ ^]^ However, we are the first to discover the expression of some of these marker genes in the amygdala; for example, Hgf exhibits a unique expression pattern and novel clustering. Further studies on these genes may help to elucidate their functions in the BLA in depth. Other marker genes, such as Moxd1, which is broadly expressed in the BLA and CeA, may be related to some functions common to these two brain regions.

Analysis of DEGs between the sham and TBI groups was based on 4 types of neurons and 5 types of nonneuronal cells. The transcriptional features of each cell type differed significantly, and the number of DEGs in neurons was the largest. KEGG analysis showed that the enrichment of different pathways differed in different cell types (Figure 3B). Our study is novel in that it provides the specific cell types in which these KEGG pathways are enriched and offers new insights into the functions of the individual cell types. Neurons were divided into 4 types of cells in this study, but it needs to be emphasized that we could not distinguish whether the nonneuronal cells were from the BLA or CeA. Therefore, in vivo experiments are needed in the future to further verify the differences in the pathways enriched in the nonneuronal cell types.

The results of FISH analysis demonstrated that Dcn is synthesized by glutamatergic neurons and significantly increased in the BLA after TBI, which is consistent with the snRNA‐seq data. Although a previous basic research study indicated that Dcn expression was increased in cerebral cortex after TBI,^[^ ^32^ ^]^ the authors suggested that Dcn has antioxidant and anti‐inflammatory activities. To the best of our knowledge, this is the first study to report that Dcn expression is upregulated in the BLA after TBI. Compared to the snRNA‐seq data, the bulk RNA‐seq data identified fewer DEGs, and because the snRNA‐seq data identified more DEGs, this technique was used to help us understand some of the potential in‐depth mechanisms.

The expression of Dcn was knocked out in BLA to verify its biological function. The amygdala is a key brain structure for processing fear and anxiety, two core symptoms of PTSD.^[^ ^33^ ^]^ Pavlovian fear conditioning test is a typical behavioral test used to assess exaggerated, dysfunctional fear and to investigate the neurobiological basis of PTSD.^[^ ^34^ ^]^ Our results showed an increase in fear conditioning response after TBI, which is widely consistent with recent studies.^[^ ^35^, ^36^ ^]^ Dcn knockout alleviated fear conditioning response caused by TBI, as observed upon chABC injection. However, anxiety behaviors in TBI were not changed after Dcn was knocked out, and we speculate that this was because anxiety assessments in rodent models of TBI exhibit substantial inconsistencies.^[^ ^37^ ^]^ These findings strongly indicate an important role for Dcn in the TBI‐associated fear conditioning response.

Because Dcn is related to the ECM, we first investigated the expression of PNNs in the BLA. Our results demonstrated that high Dcn expression could suppress the expression of PNNs. A previous study also reported PNNs to be downregulated in the brain cortex after TBI.^[^ ^17^ ^]^ PNNs in hippocampus could protect recent and remote contextual memory through regulation of parvalbumin neuron GABA release.^[^ ^38^ ^]^ Besides, PNNs in the adult sensory cortex is vital for fear learning and consolidation in response to pure tones.^[^ ^39^ ^]^ Functions of Dcn can be divided into two types:^[^ ^40^ ^]^ the first is the direct maintenance of cellular structures, such as delayed collagen fibrillogenesis; the second is outside‐in signal transduction, such as the suppression of Semaphorin 3A through erythroblastic leukemia viral oncogene homolog B4 (ErbB4) and signal transducer and activator of transcription 3 (STAT3) function.^[^ ^41^ ^]^ These studies suggest that Dcn plays an important role in inhibiting PNNs. Our IF results further showed that TBI specifically reduced the number of PNNs+CaMKII+ neurons, while chABC nonselectively reduced PNNs expression. We inferred that the enzyme chABC degrades PNNs+CaMKII+ neurons and PNNs+Gad67+ neurons nonselectively. Dcn is synthesized by excitatory neurons, which explains why Dcn knockout only decreased PNNs around CaMKII+ neurons. What's more, Dcn specifically regulated PNNs surrounding excitatory neurons, which is unlike the functions of chABC and Chst3.^[^ ^42^ ^]^

PNNs‐mediated regulation of synaptic plasticity has been widely reported,^[^ ^8^ ^]^ and the glutamate‐GABA balance is very important for maintaining normal function in the BLA.^[^ ^43^ ^]^ To investigate whether the glutamate‐GABA balance was changed in the groups, the expression levels of NR1, NR2, NR3, and Gad67 in the BLA were observed. Because NR1 is a subunit of the NMDA receptor, quantification of NR1 reflects the total number of NMDA receptors;^[^ ^44^ ^]^ NR2 and NR3 are also important subunits. Similarly, Gad67 is an enzyme that synthesizes the inhibitory neurotransmitter GABA.^[^ ^45^ ^]^ Our results demonstrated that after TBI, NR1 expression in the BLA increased, while Gad67 expression decreased, consistent with a previous study.^[^ ^7^ ^]^ Dcn knockout attenuated the high expression level of NR1 in TBI but did not change the Gad67 expression level, and similar results were obtained by TEM. In a rodent study, amygdala hyperexcitability was found to be related to chronic stress and fear conditioning.^[^ ^46^ ^]^ We speculate that an imbalance between excitation and inhibition is an important change that occurs in TBI, and Dcn knockout maintained the glutamate‐GABA balance in the BLA by regulating the expression of PNNs. That PNNs were found to regulate the glutamate‐GABA balance in a previous study is not surprising. For example, in the cortex, PNNs can change GABAergic neuron plasticity.^[^ ^38^, ^47^ ^]^ Those results demonstrates that Dcn is a novel target that specifically regulates the expression of PNNs in excitatory neurons in the BLA.

As mentioned above, in a previous study, Dcn function was divided into direct binding to collagen fibers and outside‐in signal transduction. Our IP and IF results showed a physical relationship between Dcn and Col6a3 in the BLA, which is consistent with previous studies.^[^ ^48^, ^49^ ^]^ Collagen VI is a large, triple‐helical ECM protein consisting of *α*1, *α*2, and *α*3 subunits.^[^ ^50^ ^]^ The process of collagen VI assembly is as follows: first, secreted tetramers assemble extracellularly into thin beaded filaments, and then, the beaded filaments assemble into microfibrils.^[^ ^49^ ^]^ We infer that Dcn interacts with Col6a3 and intervenes in collagen fibrillogenesis. Recently, the role of collagen VI in the brain has been gradually revealed; for example, mutations in Col6a3 could cause early‐onset isolated dystonia, and in the hippocampus, collagen VI protects neurons against amyloid‐*β* toxicity.^[^ ^51^, ^52^ ^]^ Therefore, these findings suggest that Col6a3 in the BLA is a downstream Dcn‐binding protein.

Overall, our snRNA‐seq results highlight Dcn as a natural molecule that can specifically regulate the expression of PNNs surrounding excitatory neurons in the BLA. As such, Dcn is a novel target for PTSD therapy after TBI.

## 4 Experimental Section

### Animals

Eight‐week‐old male C57BL/6J mice (20–25 g) were purchased from the Animal Center of the Fourth Military Medical University. Mice with excitatory neuron‐specific Dcn knockout were generated by stereotactic injection of AAV‐hSyn‐cre‐EGFP into the BLA of Dcn^flox/flox^ mice. All animals were housed under a 12 h light/dark cycle at a constant temperature of 23 °C with ad libitum access to food and water. All the experimental procedures were approved by the Ethics Committee of the Fourth Military Medical University (Permission Number: SYXK‐2017001). The mouse experiments were performed according to the Guidelines for the Care and Use of Laboratory Animals from the National Institutes of Health.

### Controlled Cortical Impact Model of TBI

Mice were subjected to TBI using a controlled cortical impact (CCI) device (68 099 Precision Strike; RWD, China) as previously described.^[^ ^53^ ^]^ Briefly, mice were anesthetized with 2% pentobarbital sodium and placed into a stereotaxic frame. A 3 mm craniotomy was performed at the right parietal bone (1.5 mm lateral to midline and 1.5 mm behind bregma). The tip of the 2 mm impactor piston was angled and kept perpendicular to the exposed cortical surface. The flat metal tip struck the cortex, and the following parameters were used: speed of 3 m s^−1^, depth of 1.8 mm and contact time of 200 ms. After CCI, the incision was sutured. Sham mice underwent craniotomy without CCI injury.

### Tissue Preparation

At 7 days after CCI, the mice were euthanized using an isoflurane chamber and quickly decapitated, and the brain was rapidly extracted and placed in ice‐cold artificial cerebrospinal fluid (ACSF) consisting of the following: 124 × 10^−3^ m NaCl, 2.5 × 10^−3^ m KCl, 1.2 × 10^−3^ m NaH_2_PO_4_, 24 × 10^−3^ m NaHCO_3_, 5 × 10^−3^ m HEPES, 13 × 10^−3^ m glucose, 2 × 10^−3^ m MgSO_4_, and 2 × 10^−3^ m CaCl_2_ bubbled with carbogen gas (95% O_2_ and 5% CO_2_) at pH 7.3–7.4. The brain was sectioned on a vibratome (Leica VT1000P, Germany) into 300 µm coronal slices. The amygdala region (containing the BLA and CeA) from the ipsilateral side of the TBI was microdissected under a dissecting microscope in accordance with The Mouse Brain in Stereotaxic Coordinates (the second edition) (Figure 1A). These dissected tissues were used for snRNA‐seq, bulk RNA‐seq, Western blotting and quantitative real‐time quantitative PCR (RT‐qPCR) as needed.

### Nucleus Isolation, Library Preparation, and Sequencing

Each group included 3 independent samples, and each independent sample was a mixture of amygdala tissues from 6 mice. Freshly obtained brain tissues were immediately processed for nucleus isolation, library preparation, and sequencing by Gene Denovo Biotechnology Co. Ltd. (Guangzhou, China) according to the guidelines of 10X Genomics (10X Genomics, USA). Approximately 500 mg of tissue from the amygdala of 6 mice was pooled and dissociated into a single‐nucleus suspension. The brain tissue was homogenized in ice‐cold homogenization buffer (0.25 m sucrose, 5 × 10^−3^ m CaCl_2_, 3 × 10^−3^ m MgAc_2_, 10 × 10^−3^ m Tris‐HCl (pH 8.0), 0.1 × 10^−3^ m EDTA, 1x protease inhibitor, and 1 U µL^−1^ RiboLock RNase inhibitor) with pestle strokes. Next, the homogenates were filtered through a 70 × 10^−6^ m cell strainer to collect the nuclear fraction. The nuclear fraction was mixed with an equal volume of 50% iodixanol and added on top of a 30% iodixanol solution. This solution was then centrifuged for 20 min at 10 000 ×g at 4 °C. After the myelin layer was removed from the top of the gradient, the nuclei were collected from the 30% iodixanol interface. The nuclei were resuspended in nuclear wash buffer and resuspension buffer (0.04% bovine serum albumin, 0.2 U µL^−1^ RiboLock RNase inhibitor, 500 × 10^−3^ m mannitol and 0.1 × 10^−3^ m phenylmethanesulfonyl fluoride (as a protease inhibitor) in PBS) and pelleted for 5 min at 500 ×g and 4 °C. The nuclei were filtered through a 40 × 10^−6^ m cell strainer to remove cell debris and large clumps. The nuclear concentration was manually assessed using trypan blue counterstaining and a hemocytometer. Finally, the nuclear concentration was adjusted to 700–1200 nuclei µL^−1^, and the nuclei were examined with a 10X Chromium platform. Reverse transcription, cDNA amplification and library preparation were performed based on the protocol from the manufacturer.

### Data Preprocessing

Raw reads were preprocessed using Cell Ranger (version 3.1.0) with the default parameters and aligned to the pre‐mRNA reference (Ensemble_ release 100, *Mus musculus*). For quality control, cells with gene counts between 500 and 4000 per cell, UMI counts < 8000 per cell, and a percentage of mitochondrial genes < 10% were retained for downstream analysis. Then, the global‐scaling normalization method “LogNormalize” was used to normalize the gene expression measurements for each cell by total expression, multiplied this by a scale factor (1 × 10^4^ by default), and log‐transformed the result with the following formula

(1) $$\mathrm{gene}\;\mathrm{expression}\;\mathrm{level}=\mathrm{lo}g_{10}\left(1+\left(\mathrm{UMI}\;A÷\mathrm{UMI}\;\mathrm{total}\right)\times 10^{4}\right)$$

After data integration and scaling, principal component analysis (PCA) was applied with the “RunPCA” (Seurat package) function, and appropriate principal components were selected for subsequent analysis.

### Cell Clustering

Distances between cells were calculated based on the previously identified PCA score. In brief, the purpose of this method was to embed cells in a graph structure. First, a K‐nearest neighbor (KNN) graph was used to draw edges around cells with similar gene expression patterns, and then this graph was divided into highly interconnected communities or quasicliques. Similar to PhenoGraph, a KNN graph based on the Euclidean distance in the PCA space and refined the edge weights between any two cells based on the shared overlap in their local neighborhoods (Jaccard distance) was first constructed. Then, these datasheets were visualized by employing t‐SNE, which is a powerful tool that places cells with similar local neighborhoods in high‐dimensional space together in low‐dimensional space. Analysis of DEGs between the sham and TBI groups for each cell type was performed using the “FindMarkers” (Seurat package) function by likelihood ratio test and correcting for the number of detected UMI biases. DEGs were uploaded for KEGG analysis using Metascape (http://metascape.org).

### FISH Analysis

TBI or sham mice were anesthetized with 5% isoflurane and transcardially perfused with 0.01 m PBS followed by 4% paraformaldehyde (PFA). The brains were cryopreserved in 4% PFA overnight and then incubated in 30% sucrose solution in PBS at 4 °C for 24 h. Brain slices were cut at 7 µm. RNAscope Multichannel FISH (ACD Bioscience, USA) was used to assess the expression of the genes, according to the manufacturer's instructions. Fluorescent images were captured under an A1 Si confocal microscope (Nikon, Japan).

### Bulk RNA‐seq

Similar to snRNA‐seq, each group include three independent samples, and each independent sample was a mixture of amygdala tissues from six mice. Freshly obtained brain tissues were immediately processed for RNA extraction and sequencing by Gene Denovo Biotechnology Co. Ltd. (Guangzhou, China). Briefly, total RNA was extracted using a TRIzol reagent kit (Invitrogen, USA) in accordance with the manufacturer's instructions. Oligo(dT) beads were used to enrich the eukaryotic mRNA, and a Ribo‐Zero Magnetic kit (Epicentre, USA) was used to remove rRNA to enrich the prokaryotic mRNA. Then, fragmentation buffer was used to fragment the enriched mRNA sequences into short fragments, and random primers were used to reverse transcribe the fragments into cDNA. Second‐strand cDNA was synthesized with DNA polymerase I, RNase H, dNTPs, and buffer. A QiaQuick PCR extraction kit (Qiagen, the Netherlands) was used to purify the cDNA fragments, repair the ends, add poly(A), and connect to the Illumina sequencing adapter. The ligation product was selected by agarose gel electrophoresis based on size, subjected to PCR amplification and sequenced using a NovaSeq 6000 (Illumina, USA).

### RT‐qPCR

Total RNA was extracted from tissues with TRIzol reagent (Invitrogen, USA) following the manufacturer's protocol. Total RNA (1 mg) was reverse transcribed to generate cDNA with HiScript II Q RT SuperMix for RT‐qPCR (+gDNA Wiper) (Takara, Japan). The primer sequences are listed in Table S1 (Supporting Information). RT‐qPCR was performed on the iQTM 5 Optical Module Real‐Time PCR Detection System (Bio‐Rad, China) using a SYBR Premix Ex Taq II kit (Takara, Japan). The 2‐ΔΔCT method was used to quantitate changes in relative gene expression normalized to *β*‐actin.

### IF Analysis

Mice were anesthetized with 2% pentobarbital and perfused with 0.01 m PBS, followed by fixation with 4% PFA. Coronal brain sections (30 µm) of the amygdala were incubated with PBS containing 0.1% Triton X‐100 for 20 min and blocked with PBS containing 5% goat serum (Gibco, USA) for 60 min. Then, the samples were incubated with the following antibodies and reagents: anti‐Dcn (1:500, Abcam, USA), anti‐CaMKII (1:500, Millipore, USA), anti‐Iba1(1:500, Abcam, USA), anti‐GFAP(1:500, Abcam, USA), biotinylated *Wisteria floribunda* lectin (1:1000, Sigma, USA), anti‐Gad67 (1:500, GeneTex, USA), anti‐Col6a3 (1:100, Santa Cruz, USA), streptavidin‐Alexa Fluor 594 (1:500, Abcam, USA), and antimouse/rabbit Alexa Fluor 594/488 (1:500, Invitrogen, USA). Images were acquired using an A1 Si confocal microscope (Nikon, Japan).

### Virus and chABC Injection

AAV2/9‐hSyn‐cre‐EGFP or AAV2/9‐hSyn‐EGFP was purchased from Hanbio Technology Co. Ltd. (Shanghai, China) and delivered (0.5 µL at 0.05 µL min^−1^) into the right BLA of Dcn ^flox/flox^ mice at the following coordinates: 1.7 mm posterior to bregma, 3.3 mm lateral to midline, and 4.0 mm below the cortical surface. In this study, 14 days after virus injection, the mice underwent CCI, and 21 days after virus injection, the mice were used for the following experiments: RT‐qPCR, Western blotting, and behavioral tests (Figure 5A). 25 mU of chABC was dissolved in 0.5 µL of 0.1 m PBS and delivered (0.05 µL min^−1^) into the right BLA of C57BL/6J mice at the coordinates as mentioned above; the mice were used for further analyses after 7 days.

### FCR Test

The contextual and cued fear conditioning paradigms included one training session and two tests, as previously described^[^ ^54^ ^]^ (Figure 6A). Seven days after TBI, the mice were individually placed in the contextual chamber (242×242×300 mm) for 180 s of adaptation and then presented with a pure tone (28 s, 1 kHz, 90 dB), followed by a foot shock (2 s, 1.0 mA). Tone‐foot‐shock pairing was repeated 3 times, and the mice then rested in the chamber for 2 min. Twenty‐four hours later, the mice were subjected to the contextual fear response test and placed into the contextual chamber for 300 s without exposure to tone or foot shock. After 1 h, the mice were placed in the neutral chamber for 180 s with a neutral tone (4 kHz, 80 dB, sine wave) but without foot shock. An automated analytical system (Vanbi, China) recorded the activities of all mice. Freezing was defined as the detection of no movement from the mouse for 2 s.

### EPM Test

The Plexiglas apparatus consisted of two opposite‐facing “open” arms (50 mm (D) x 250 mm (W)), two opposite‐facing “closed” arms (50 mm (D) x 250 mm (W) x 50 mm (H)), and a central area (50 mm (D) x 50 mm (W)). This apparatus was raised 50 cm above the ground. At the beginning of the test, mice were placed alone in the central area facing a closed arm and allowed to freely explore the maze for 5 min. A video camera tracked the motions of each mouse, and the times spent in the open and closed arms were calculated by EthoVision XT Version 8.0 (Noldus, the Netherlands).

### OF Test

The open field apparatus is a box with an open top, and the parameters are 432 mm (D) x 432 mm (W) x 305 mm (H). At the beginning of the test, mice were placed alone in the center of the open field and allowed to freely explore the field for 10 min. The central zone was defined by the zones from (40, 40) to (130, 130). A video camera recorded the motions of each mouse, and the times spent in the central zone were calculated by EthoVision XT Version 8.0 (Noldus, the Netherlands).

### Western Blotting Analysis

Western blotting was performed in accordance with standard procedures (Molecular Clone, Edition II). BLA tissues were collected as described above and homogenized in ice‐cold lysis buffer containing phosphatase and protease inhibitors. Twenty micrograms of total protein from the tissues was resolved by SDS‐PAGE and transferred to PVDF membranes (Millipore, USA). After blocking with 5% nonfat milk, the following primary antibodies were applied: anti‐NR1 (1:3000, Millipore, USA), anti‐Gad67 (1:1000, GeneTex, USA), anti‐NR2A(1:500, Proteintech, China), anti‐NR2B(1:500, Proteintech, China), anti‐NR3A(1:500, Invitrogen, USA), anti‐NR3B(1:500, Invitrogen, USA), anti‐Dcn (1:1000, Abcam, USA), anti‐Col6a3 (1:500, Santa Cruz, USA), anti‐Col17a1 (1:1000, Invitrogen, USA), anti‐*β*‐actin (1:5000, Cell Signaling, USA), and antimouse/rabbit secondary antibodies (1:5000, ABclonal, China). Images were captured using a Bio‐Rad imaging system (Bio‐Rad, China), and the bands were analyzed by ImageJ (Version 1.53C).

### TEM

Mice were deeply anesthetized and transcardially perfused with 4% PFA in 0.01 As previously described, BLA tissues were fixed in 4% glutaraldehyde at 4 °C for 16 h. After fixation in 1% osmium tetroxide for 60 min, the samples were dehydrated using a graded ethanol series and embedded in resin. An ultramicrotome was used to trim and cut the embedded sample blocks, after which the slices were placed on 200‐slot grids coated with polyvinyl alcohol ester and imaged under a JEM‐1400 electron microscope (HITACHI, Japan).

### Immunoprecipitation (IP)

HT22 cells (ProCell, China) were collected and lysed with IP lysis buffer (Roche, Switzerland) at 4 °C for 1 h. The lysates were cleared by centrifugation (13 000 rpm, 10 min). The supernatant (1000 µg) was immunoprecipitated with 20 µg of anti‐Dcn (1:50, Thermo Fisher Scientific, USA) or anti‐Col6a3 (1:50, Thermo Fisher Scientific, USA) overnight with gentle inversion at 4 °C. Subsequently, 40 µL of fully suspended protein A/G magnetic beads (MedChemExpress, China) was added, and the mixture was incubated for 2 h at room temperature. Complexes that bound the protein A/G conjugate were washed, resolved in SDS‐PAGE loading buffer, and subjected to analysis by Western blotting or LC–MS/MS analysis.

### LC–MS/MS Analysis of Proteins

After Coomassie Blue Fast staining of SDS–PAGE gels, bands from the gels were sent to Biotree Biomedical Technology Co. Ltd. (Shanghai, China) for LC–MS/MS analysis. Briefly, the analysis was performed on a Q Exactive HFX Orbitrap instrument (Thermo Fisher Scientific, USA) with a nanoelectrospray ion source. The data‐dependent top20 acquisition method was used to dynamically choose the most abundant precursor ions from the survey scan (350–1600 mz^−1^) for higher energy collisional dissociation fragmentation. MS files were processed with Proteome Discoverer software (version 2.4.0.305; Thermo Fisher Scientific, USA) and the built‐in Sequest HT search engine.

### Statistical Analyses

Statistical analyses were performed with SPSS 21.0 software. All the data are expressed as the mean ± SEM (standard error of the mean). Two groups were compared using unpaired two‐tailed Student's test, and one‐way analysis of variance (ANOVA) was employed to compare multiple groups. Bonferroni post hoc tests were applied to detect statistically significant differences between groups. Differences for which *p* < 0.05 were considered statistically significant.

## Conflict of Interest

The authors declare no conflict of interest.

## Supporting information

Supporting Information

## Data Availability

The snRNA‐seq and bulk RNA‐seq data generated in the current study are available under accession numbers PRJNA739209 (https://dataview.ncbi.nlm.nih.gov/object/PRJNA739209?reviewer=lok0rsh77u672ld7vh0h2afbj8) and PRJNA758270 (https://dataview.ncbi.nlm.nih.gov/object/PRJNA758270?reviewer=htskujti8ru0qunk72ggr604dl). All the codes used in this study are available upon reasonable request.
