# Supplementary material for: Single‐Nucleus RNA Sequencing Reveals that Decorin Expression in the Amygdala Regulates Perineuronal Nets Expression and Fear Conditioning Response after Traumatic Brain Injury
Source: Adv Sci (Weinh). 2022 Jan 17;9(7):2104112. doi: 10.1002/advs.202104112 (PMC8895134; doi:10.1002/advs.202104112)
Supplement: Supplementary file 1 — Supporting Information [file ADVS-9-2104112-s001.pdf]

ADVANCED SCIENCE
Open Access

Supporting Information

for *Adv. Sci.,* DOI: 10.1002/advs.202104112

Single-Nucleus RNA Sequencing Reveals that Decorin Expression in the Amygdala Regulates Perineuronal Nets Expression and Fear Conditioning Response after Traumatic Brain Injury

Yingwu Shi, Xun Wu, Jinpeng Zhou, Wenxing Cui, Jin Wang, Qing Hu, Shenghao Zhang, Liying Han, Meixuan Zhou, Jianing Luo, Qiang Wang, Haixiao Liu, Dayun Feng, Shunnan Ge *, *Yan Qu* *

Supporting Information

Single-Nucleus RNA Sequencing Reveals that Decorin Expression in the Amygdala Regulates Perineuronal Nets Expression and Fear Conditioning Response after Traumatic Brain Injury

*Yingwu Shi, Xun Wu, Jinpeng Zhou, Wenxing Cui, Jin Wang, Qing Hu, Shenghao Zhang, Liying Han, Meixuan Zhou, Jianing Luo, Qiang Wang, Haixiao Liu, Dayun Feng, Shunnan Ge *, Yan Qu **

Supporting Information includes:

Figure S1-S6

Table S1-S2

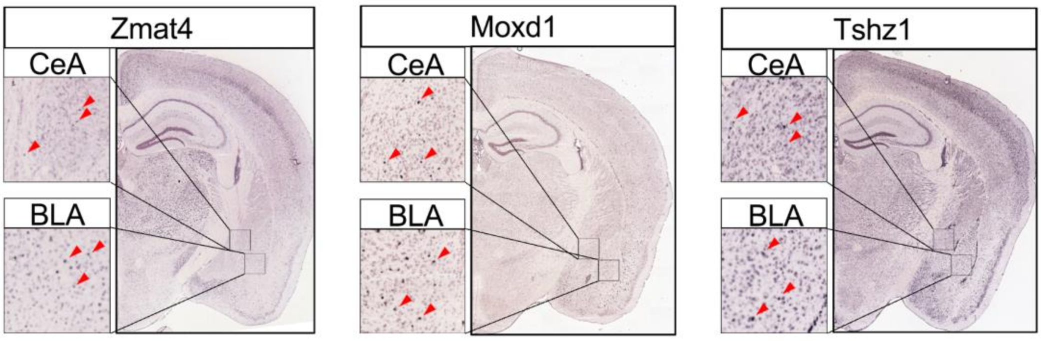

Figure S1. related to Figure 2

Results of the RNA ISH analysis of marker genes that are expressed in both the BLA and CeA.

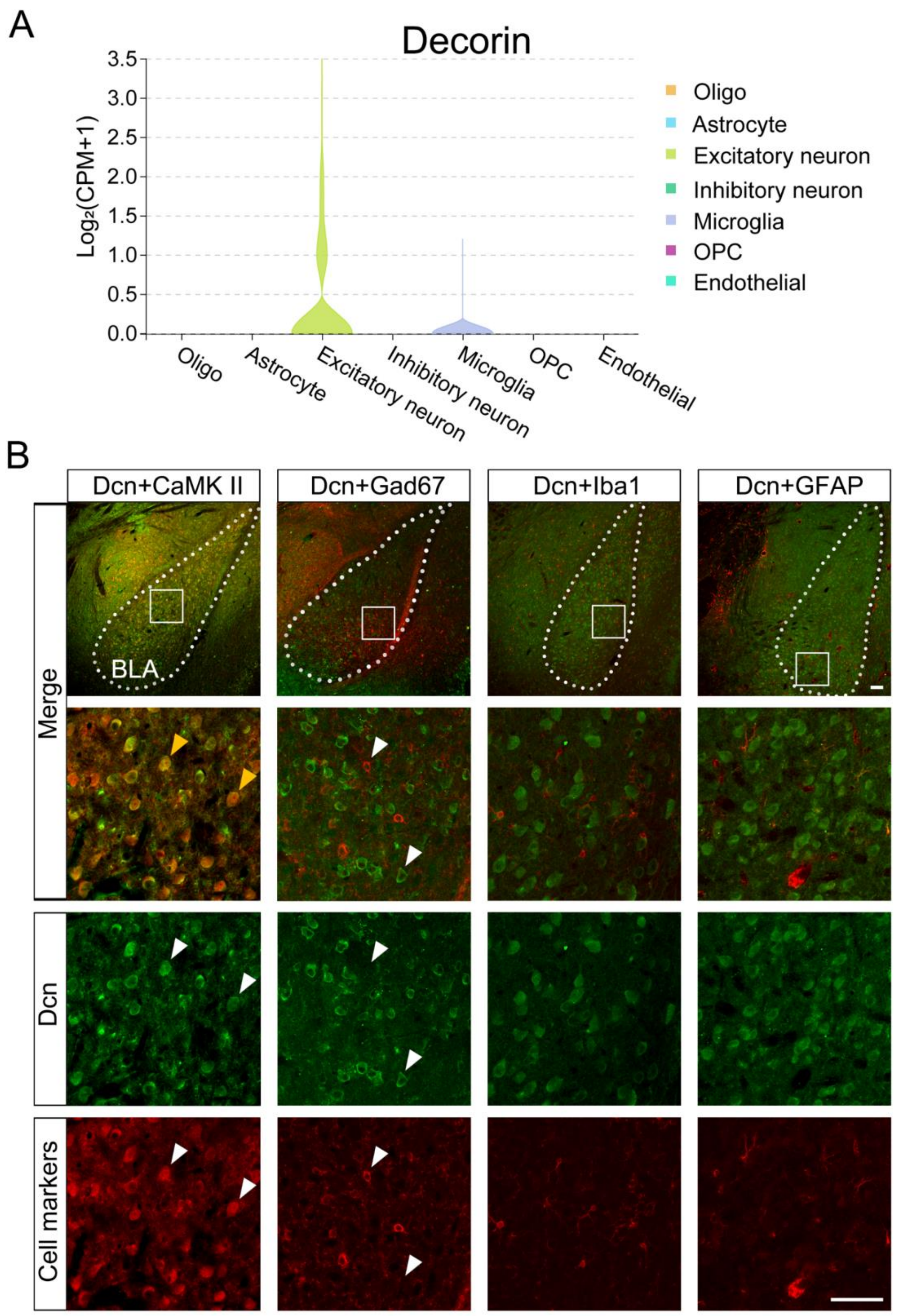

Figure S2, related to Figure 3

Expression pattern of Dcn in the amygdala. (A) Violin plot showing the expression of Dcn in the different cell types. (B) Double-labeling immunofluorescence of Dcn and cell markers (CaMKII, Gad67, Iba1 and GFAP). Scale bar, 100 μm;

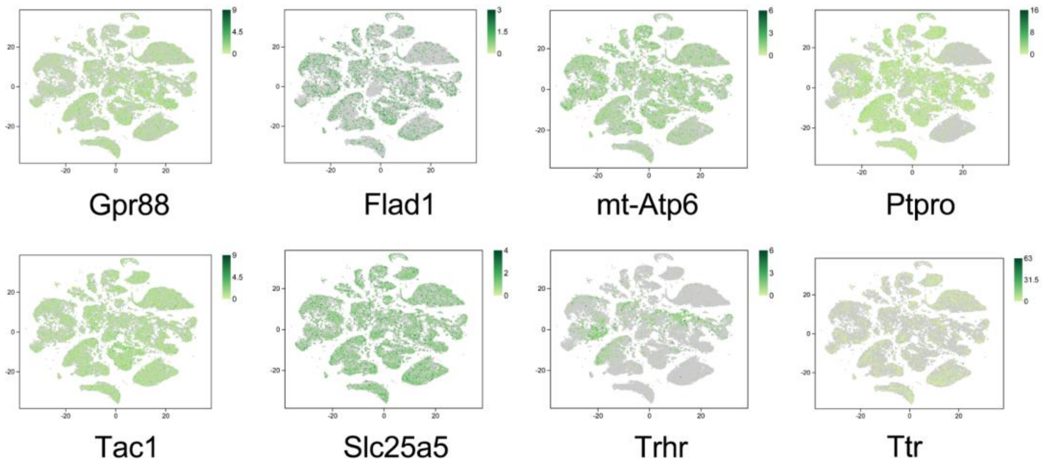

Figure S3, related to Figure 3

t-SNE plot showing the DEGs widely expressed in more than one cell type.

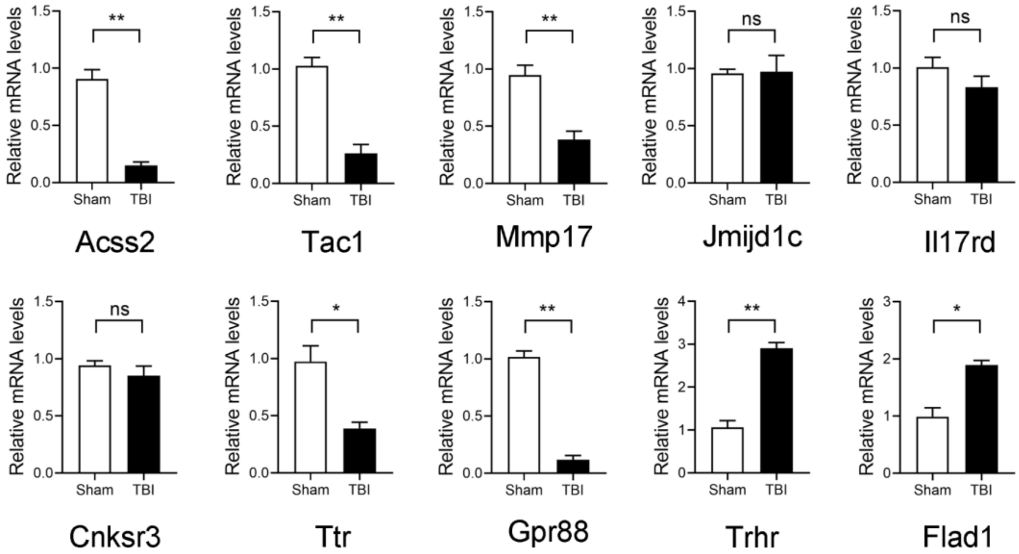

Figure S4, related to Figure 3

RT-qPCR validation of the DEG expression of the TBI and sham groups. Data are presented as the mean ± SEM, n=3, ** p<0.01, * p<0.05 (t test).

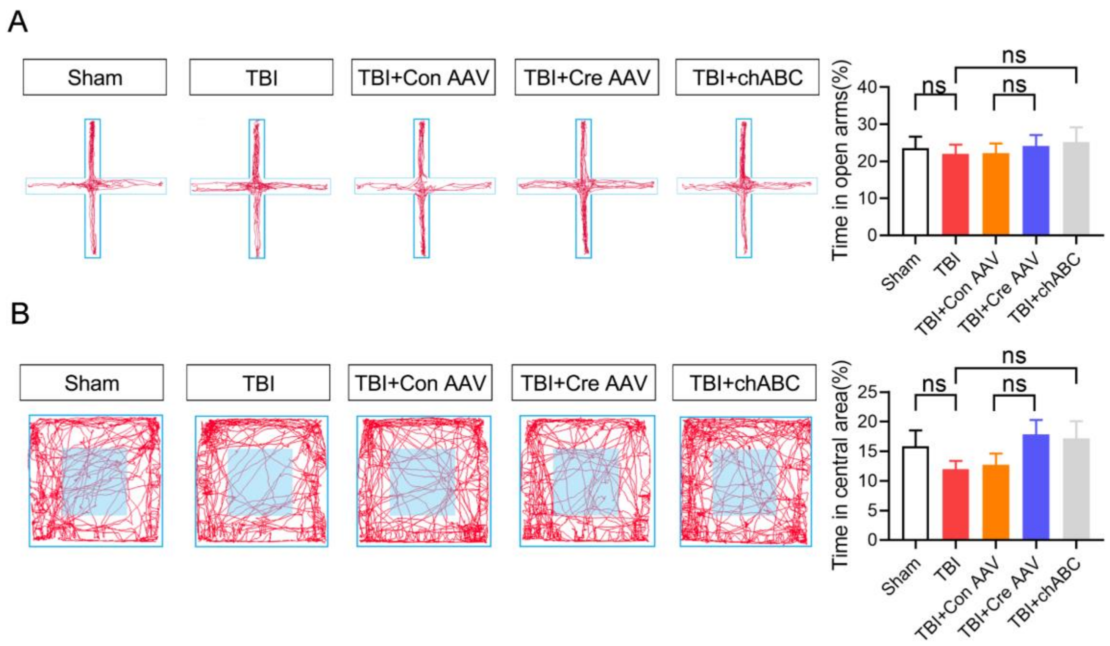

Figure S5, related to Figure 5

Representative OF and EZM images and analysis of the 5 groups. Data are presented as the mean ± SEM, n=6, ** $p<0.01$ (one-way ANOVA with Bonferroni's post hoc test).

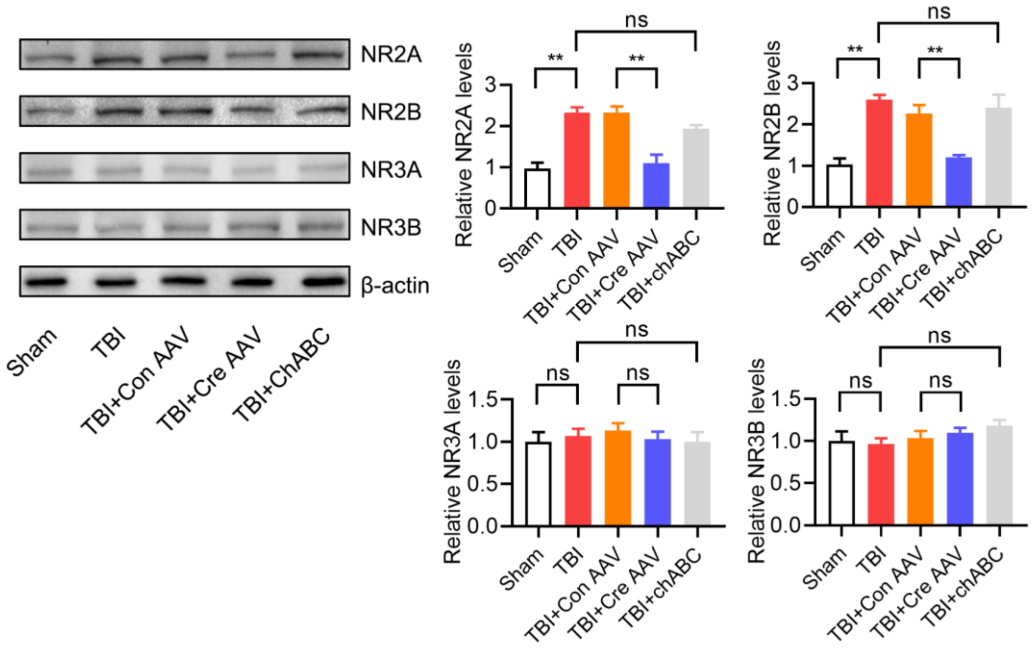

Figure S6, related to Figure 6

Western blotting analysis showing of the expression of NR2(NR2A, NR2B) and NR3(NR3A, NR3B) in the BLA tissues. Data are presented as the mean ± SEM, n=6, ** $p<0.01$ (one-way ANOVA with Bonferroni's post hoc test).

Table S1. Top 5 DEGs of each cell types

Cell types	Top 5 ranked DEGs
Astrocyte	Acss2, Tac1, Gpr88, Gfap, Adcy5
Microglia	Tac1, Mmp17, Rad51b, Chil1, Nat14
Endothelial	Jmjd1c, Il17rd, Srgap1, Tshz2, Ankfy1
Oligo	Cnksr3, Tac1, Frmd4a, Cemip2, Ptprb
OPC	Ttr, Gpr88, Tle2, Hpca, Dhfr
BLA-Exc	Dcn, Trhr, Ptpro, Arhgap6, Kirrel3
BLA-Inhib	Gpr88, Ttr, mt-Atp6, Fgf1, Cryab
CeA-Inhib	Tac1, Ttr, Slc25a5, Adra1a, Mid1
CeA/BLA-Inhib	Ttr, Flad1, Gpr88, Grap2, Ptgds

Table S2 The sequence of Primers

Genes name	Forward (5’- 3’)	Reverse (5’- 3’)
Acss2	GTTCTGCTTCTTTCCCATTCTTC	GCTGCTTGAACACCAGATAACC
Tac1	TCGATGCCAACGATGATCTAAA	AACTGCTGAGGCTTGGGT
Mmp17	ACCTTCCGTTCCTCAGATGC	CATAGGGAAGTCCATAGCGTAGC
Jmijd1c	TTCAGATACGACAACTGTACCACCTA	CTGAGATTCCATTCCAGTCCTTC
Il17rd	ATTGTCCCTTTCCCTTCCATTA	TATTCTGGTCCTGCCTGCAAGT
Cnksr3	TCCAGAAGGATTGCCTCATAGC	CATGCAGTCCGTCGTAGGTTG
Ttr	TGGTCAAAGTCCTGGATGCTGT	TGCGATGGTGTAGTGGCGAT
Dcn	AAGGCTTTACCTGTCTAAGAACCA	GGTGTCTGAGATGCGAATGTATG
Gpr88	AACTCCTCCTCCACTTCGACCT	CATTCACGATGAAGGCGTTG
Trhr	AGGAAGCAGGTCACCAAGATG	GGTTGATGGCACTGTTGAGATAA
Flad1	ACTCCTGTAGCCTCAGCCATTT	GGTATGCTGGACGGTAGACAGG
β-actin	GTGACGTTGACATCCGTAAAGA	GTAACAGTCCGCCTAGAAGCAC
